# Association of treatments for acute myocardial infarction and survival for seven common comorbidity states: a nationwide cohort study

**DOI:** 10.1186/s12916-020-01689-5

**Published:** 2020-08-24

**Authors:** Mohammad E. Yadegarfar, Chris P. Gale, Tatendashe B. Dondo, Chris G. Wilkinson, Martin R. Cowie, Marlous Hall

**Affiliations:** 1 Leeds Institute of Cardiovascular and Metabolic Medicine, University of Leeds, Leeds, UK; 2 Leeds Institute for Data Analytics, University of Leeds, Worsley Building, Level 11, Clarendon Way, Leeds, LS2 9NL UK; 3 Department of Cardiology, Leeds Teaching Hospitals NHS Trust, Leeds, UK; 4 Population Health Sciences Institute, Newcastle University, Newcastle upon Tyne, UK; 5 Faculty of Medicine, National Heart & Lung Institute, Imperial College London, London, UK; 6 Royal Brompton and Harefield NHS Foundation Trust, London, UK

**Keywords:** Acute myocardial infarction, Comorbidity, Survival, Guideline care

## Abstract

**Background:**

Comorbidity is common and has a substantial negative impact on the prognosis of patients with acute myocardial infarction (AMI). Whilst receipt of guideline-indicated treatment for AMI is associated with improved prognosis, the extent to which comorbidities influence treatment provision its efficacy is unknown. Therefore, we investigated the association between treatment provision for AMI and survival for seven common comorbidities.

**Methods:**

We used data of 693,388 AMI patients recorded in the Myocardial Ischaemia National Audit Project (MINAP), 2003–2013. We investigated the association between comorbidities and receipt of optimal care for AMI (receipt of all eligible guideline-indicated treatments), and the effect of receipt of optimal care for comorbid AMI patients on long-term survival using flexible parametric survival models.

**Results:**

A total of 412,809 [59.5%] patients with AMI had at least one comorbidity, including hypertension (302,388 [48.7%]), diabetes (122,228 [19.4%]), chronic obstructive pulmonary disease (COPD, 89,221 [14.9%]), cerebrovascular disease (51,883 [8.6%]), chronic heart failure (33,813 [5.6%]), chronic renal failure (31,029 [5.0%]) and peripheral vascular disease (27,627 [4.6%]).

Receipt of optimal care was associated with greatest survival benefit for patients without comorbidities (HR 0.53, 95% CI 0.51–0.56) followed by patients with hypertension (HR 0.60, 95% CI 0.58–0.62), diabetes (HR 0.83, 95% CI 0.80–0.87), peripheral vascular disease (HR 0.85, 95% CI 0.79–0.91), renal failure (HR 0.89, 95% CI 0.84–0.94) and COPD (HR 0.90, 95% CI 0.87–0.94). For patients with heart failure and cerebrovascular disease, optimal care for AMI was not associated with improved survival.

**Conclusions:**

Overall, guideline-indicated care was associated with improved long-term survival. However, this was not the case in AMI patients with concomitant heart failure or cerebrovascular disease. There is therefore a need for novel treatments to improve outcomes for AMI patients with pre-existing heart failure or cerebrovascular disease.

## Background

Comorbidity is common and has a substantial negative impact on the prognosis of patients with acute myocardial infarction (AMI) [1–3]. Receipt of guideline-indicated treatments for AMI is associated with improved survival [4–6], reduced morbidity [6] and lower subsequent healthcare expenditure [7]. However, there is little information about the impact of comorbidities on the receipt of treatment for AMI and, in particular, the impact of these treatments on prognosis for a range of comorbidities. Over 100,000 people in the United Kingdom (UK) were admitted to hospital with AMI in 2017/2018 [8], and of these, 59.5% have comorbidities [1]. It is therefore necessary to identify comorbidity states where receipt of treatment is suboptimal, or where AMI treatment has little impact on prognosis in order to guide research into novel therapies, thereby optimising patient-centred care delivery.

Whilst there is good evidence to suggest that comorbidity adversely influences treatment pathways for a number of specific diseases including diabetes, cancer and chronic obstructive pulmonary disease (COPD) [3, 9–12], there is a paucity of information about the impact of comorbidity on treatment delivery and treatment efficacy for AMI. For example, it has been shown that patients with cancer who present with AMI are less likely to receive percutaneous coronary intervention (PCI), P_2_Y_12_ inhibitors and statin therapy [13]; those with COPD and AMI are less likely to receive beta-blockers [14]; and those with mental health disorders and AMI are less likely to receive reperfusion therapy [15]. Patients with AMI and diabetes have reduced survival at 30 days and 1 year [16–18]. Notably, the evidence to date is limited to studies of the impact of a single comorbidity on the treatment for AMI [13–15], and based on either small-scale single centre data with long-term follow-up [14] or large-scale multicentre data with outcomes limited to in-hospital or short-term mortality [13, 15]. Moreover, to our knowledge, there is no evidence to date concerning the efficacy of AMI treatments on clinical outcomes for patients with AMI and comorbidity.

The UK is one of only two countries worldwide that has a continuous acute coronary syndrome clinical registry that includes all hospitals in its nationwide health service and has detailed information about patient and treatment characteristics—the Myocardial Ischaemia National Audit Project (MINAP). MINAP provides a unique opportunity to undertake high resolution phenotype-specific interrogation of comorbidity, treatment pathways and long-term survival for patients with AMI in a nationwide cohort. In the absence of previous robust evidence, our objectives in this study were to assess both the effect of comorbidity states on receipt of guideline-indicated AMI care, and the impact of receipt of care on long-term survival outcomes in the presence of comorbidity.

## Methods

### Study design and participants

Data were obtained from MINAP, a comprehensive national clinical registry of patients hospitalised with AMI which includes 130 data fields collected during AMI patients’ treatment course from the first contact, throughout hospitalisation, to discharge and rehabilitation [19, 20]. Submission of data to MINAP is mandated by the Department of Health for all hospitals in England and Wales. Data are collected prospectively at each hospital, electronically encrypted and transferred online to a central database. Data entry is subject to routine error checking and an annual data validation exercise. Mortality data are obtained through linkage with the Office for National Statistics death records. Further details of MINAP have been published elsewhere [19–22].

**Fig. 1:**
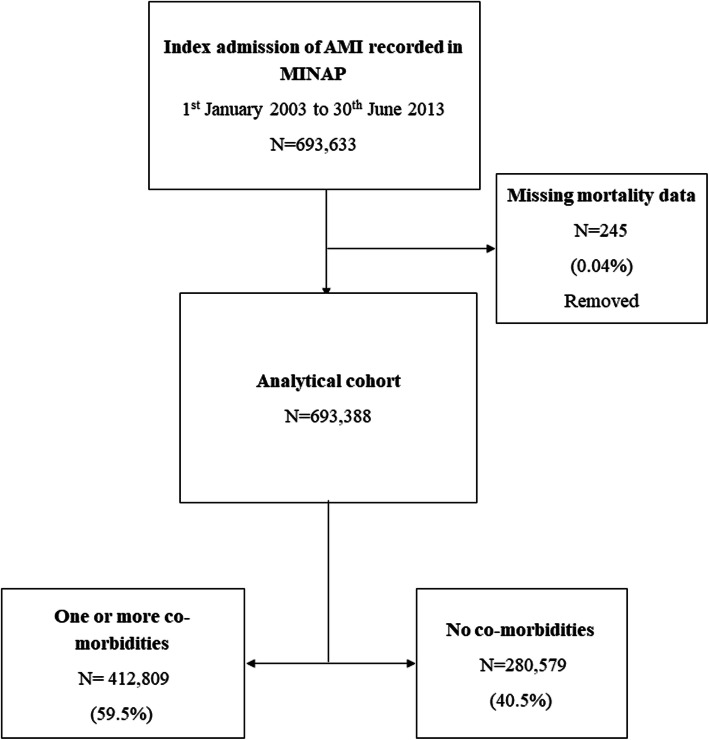
Derivation of the analytical cohort

The analytical cohort included 693,388 patients aged 18 years and over who were admitted to hospital with AMI between January 1, 2003, and June 30, 2013 (Fig. 1) [1]. The National Institute for Cardiovascular Outcomes Research (NICOR) which includes the MINAP database (ref: NIGB: ECC 1-06 (d)/2011) has support under section 251 of the National Health Service Act 2006 to use patient information for medical research without informed consent. Ethical approval for this study was not required under current National Health Service (NHS) research governance arrangements for the secondary use of anonymised patient data collected during the course of normal care.

In order to ensure accuracy of AMI treatment effects on patients, only index admission data was used in this study. Patients with a history of any of the following conditions were considered to have comorbidity: diabetes mellitus, COPD or asthma, hypertension, chronic heart failure, chronic renal failure, cerebrovascular disease and peripheral vascular disease. Guideline-indicated treatment was determined according to previously published work that mapped MINAP variables to the relevant international guidelines for the management of AMI [22]. This included pharmacological therapies (aspirin prior to, and at, admission; β-blockers, statins, angiotensin-converting enzyme inhibitors [ACEi] or angiotensin receptor blockers [ARB], P2Y_12_ inhibitors and aldosterone antagonists at discharge), non-invasive treatment (electrocardiogram, echocardiography, cardiac rehabilitation, smoking cessation advice, dietary advice) and invasive treatment (early invasive coronary procedures (reperfusion within 12 h for ST-elevation myocardial infarction (STEMI)), and an invasive coronary strategy (coronary angiography, PCI or coronary artery bypass graft (CABG) surgery) for non-STEMI (NSTEMI)) [23]. Treatment receipt was objectively recorded, with the exception of cardiac rehabilitation—which is accessed through a post-discharge referral, and is therefore a proxy measure for its receipt. Patients were classified as ineligible for a treatment if it was recorded within MINAP as contraindicated, not indicated or not applicable; if the patient declined treatment; or if the patient was hospitalised prior to the year in which the guideline treatment recommendation was published. Full eligibility criteria for therapy can be found in Additional file 1: Table S1. In addition, the following patient-level demographic data (sex, index of multiple deprivation (IMD) score—a marker for socioeconomic status), Global Registry of Acute Coronary Events (GRACE) risk score (including age, cardiac arrest, ST-segment deviation, elevated enzyme levels, systolic blood pressure, heart rate, loop diuretic [substituted for Killip class] and creatinine) and clinical characteristics (total cholesterol, discharge diagnosis (STEMI, NSTEMI), previous CABG surgery, previous PCI) were extracted from MINAP.

### Patient and public involvement

Patient and public involvement (PPI) representatives were consulted at the research design stage by providing insights into the burden of comorbidity and treatments from a patients’ perspective. They noted it was important for care provision to be monitored more closely for people with long-term health conditions, and were keen to see the outputs of proposed research. It was difficult to involve patients in other areas of the study due to data protection restrictions and the very technical methods required to analyse this large-scale health data. However, the database used in the study is managed by NICOR who work closely with PPI representatives and were commended for their excellence in PPI engagement in clinical audits by the Healthcare Quality Improvement Partnership (HQIP). PPI representatives were keen to stress that they feel information leaflets on the wards were the best way to disseminate our research findings directly to the relevant patient groups, and they will co-design such a leaflet containing lay summaries for dissemination to their peers.

### Statistical analysis

Treatment eligibility and receipt of each of the 13 guideline-indicated therapies as outlined above were calculated based on respective European Society of Cardiology guidelines for management of AMI, and mapped to MINAP data. A cumulative treatment score of treatments received out of total treatments for which the patient was eligible was derived [21]. Optimal care was defined as receipt of all treatments for which an individual was eligible (maximum score 13). Baseline characteristics were described according to each comorbidity using numbers and percentages for categorical data, means and standard deviation for normally distributed data and median and interquartile ranges for non-normally distributed data. A cumulative treatment score was calculated for patients with no comorbidity, for each comorbidity individually and for any comorbidity. The proportion with a receipt rate of at least 80% of all eligible treatments was also reported [24].

Multiple imputation by chained equations was used to create ten imputations each, with 20 iterations based on a comprehensive set of all analyses variables and auxiliary variables in order to minimise potential bias due to missing data [1]. All models presented in the main body of the paper represent pooled estimates with accompanying 95% confidence intervals according to Rubin’s rules across ten imputed datasets [25]. Full imputation model specification and a sensitivity analysis comparing results to a complete case analysis have been reported in Additional file 1: TableS2, S8-S11.

A series of logistic regression models were fitted to determine the association between the presence of each comorbidity on the receipt of optimal care for AMI (an all-or-none treatment approach). To validate this all-or-none approach, Poisson regression models were performed using the cumulative treatment score. For each model, we present unadjusted results alongside those adjusted for patient demographics (age, sex and IMD score) and a final fully adjusted model including GRACE risk score, year of diagnosis, smoking status, IMD score and each of the seven comorbidities.

To determine the combined association of comorbidities and treatment on survival, a series of Royston-Parmar flexible parametric survival models were fitted using interaction terms which included four possible exposures: those without a pre-specified comorbidity who received suboptimal care, those with the comorbidity who received suboptimal care, those without comorbidity who received optimal care and patients with comorbidity who received optimal care. For each survival model, we present unadjusted results alongside those adjusted for patient demographics (age, sex and IMD score) and a final fully adjusted model including GRACE risk score, year of diagnosis, smoking status, IMD score and each of the seven comorbidities. Royston-Parmar flexible parametric survival models were chosen in favour of standard Cox proportional hazards models for which the assumptions were not met. Selection of the scale (hazard, odds, normal or theta) and the complexity (number of degrees of freedom) of the models were informed by minimising Akaike and Bayesian information criteria.

Sensitivity analyses were conducted to determine the combined association of each respective comorbidity and treatment on survival for those patients who had a single comorbidity compared with those in whom no comorbidities were recorded. All tests were 2-sided, and statistical significance was considered as *P* < 0.05. Statistical analyses were performed in Stata IC version 14.2 and R version 3.4.3.

## Results

There were 693,388 patients with AMI (274,220 [39.6%] STEMI; 419,168 [60.5%] NSTEMI) with no comorbidities recorded in 280,579 (40.5%) and at least one comorbidity in the remainder (412,809 [59.5%]) (Fig. 1). Of this analytical cohort (median age 70.7 [IQR 59.4 to 80.1] years, 238,569 [34.5%] women), the median survival time was 2.25 years (IQR 0.88 to 4.00); 1,872,468 total person-years follow-up. The most prevalent comorbidity was hypertension (302,388 [43.6%]), followed by diabetes mellitus (122,228 [17.6%]), COPD or asthma (89,221 [12.9%]), cerebrovascular disease (51,883 [7.5%]), chronic heart failure (33,813 [4.9%]), renal failure (31,029 [4.5%]) and peripheral vascular disease (27,627 [4.0%]) (Table 1). Those without comorbidities were younger (median age 65.2 years [IQR 54.8 to 76.9]) of whom most were men (198,279 [70.9%]) compared to patients with comorbidities (Table 1). Across all comorbidities, more than two thirds of AMIs were NSTEMI, whereas amongst those without comorbidities, only half were NSTEMI (141,185 [50.3%]) (Table 1).

**Table 1:** Baseline characteristics for patients with AMI by morbidity

Number of cases	Diabetes (122,228)	COPD (89,221)	Hypertension (302,388)	Chronic heart failure (33,813)	Chronic renal failure (31,029)	Cerebrovascular disease (51,883)	Peripheral vascular disease (27,627)	No comorbidity (198,203)	All (693,388)	Missing, *N* (%)
**Age (years)**	72.7 (63–80)	73.5 (64–81)	73.4 (63.1–81.5)	80.4 (73–86)	79 (71.3–85)	78.4 (70.5–84.7)	74.6 (66–81.4)	65.2 (54.8–76.9)	70.7 (59.4–80.1)	0.1 (1025)
**Sex (female)**	36.1 (44,063)	41.2 (36,709)	38.9 (117,421)	44.6 (15,062)	37.1 (11,507)	41.1 (21,277)	31.2 (8607)	29.1 (81,270)	34.5 (238,569)	0.3 (1923)
**Smoker**										9.4 (65,000)
**Never**	39.8 (44,974)	27.2 (22,837)	39.5 (111,727)	43.0 (12,979)	42.8 (11,945)	40.5 (18,978)	25.4 (6527)	37.1 (90,868)	37.8 (237,432)	
**Ex/current**	60.2 (68,147)	72.8 (61,126)	60.5 (171,255)	57.0 (17,179)	57.2 (15,958)	59.5 (27,904)	74.6 (19,192)	62.9 (153,836)	62.2 (390,956)	
**Year**										0.0 (0)
**2003–2006**	29.8 (36,462)	32.1 (28,670)	31.1 (94,080)	36.2 (12,239)	23.7 (7343)	32.2 (16,702)	35.6 (9826)	39.9 (112,069)	35.3 (244,499)	
**2007–2010**	41.5 (50,748)	40.8 (36,432)	41.7 (126,222)	38.8 (13,110)	44.1 (13,681)	41.3 (21,416)	39.3 (10,852)	37.1 (103,980)	39.5 (274,085)	
**2011–2013**	28.7 (35,018)	27.0 (24,119)	27.2 (82,086)	25.0 (8464)	32.2 (10,005)	26.5 (13,765)	25.2 (6949)	23.0 (64,530)	25.2 (174,804)	
**IMD score**	20.3 (11.7–34.8)	21.2 (12.1–36.2)	18.0 (10.4–31.3)	18.8 (11.1–32.5)	18.6 (10.8–32.1)	18.9 (10.9–33.1)	20.0 (11.5–34.6)	18.4 (10.7–32.1)	18.3 (10.6–31.8)	8.2 (56,733)
**Systolic blood pressure (mmHg)**	139 (120–159)	137 (119–157)	141 (121–161)	133 (114–154)	136 (116–157)	138 (118–158)	137 (118–158)	136 (119–154)	21.6 (14.9–27)	19.3 (133,701)
**Heart rate (bpm)**	83 (70–99)	84 (70–100)	80 (67–94)	87 (72–104)	83 (70–98)	82 (69–98)	82 (70–98)	75 (64–90)	79 (66–93)	19.0 (131,708)
**Cholesterol (mmol/l)**	4.2 (3.4–5)	4.7 (3.9–5.7)	4.6 (3.8–5.6)	4.1 (3.4–5)	4 (3.3–5)	4.2 (3.5–5.1)	4.2 (3.5–5.2)	5.30 (4.4–6.2)	4.9 (4–5.9)	35.7 (247,700)
**Creatinine (μmol/l)**	99 (80–132)	92 (75–116)	95 (78–119)	117 (91–157)	168 (124–241)	102 (82–133)	103 (82–139)	87 (74–102)	91 (76–112)	44.5 (308,897)
**AMI diagnosis**										0.0 (0)
**STEMI**	27.8 (33,993)	30.3 (27,013)	33.2 (100,356)	14.8 (5013)	18.4 (5712)	25.3 (13,146)	25.8 (7115)	49.7 (139,394)	39.6 (274,220)	
**NSTEMI**	72.2 (88,235)	69.7 (62,208)	66.8 (202,032)	85.2 (28,800)	81.6 (25,317)	74.7 (38,737)	74.3 (20,512)	50.3 (141,185)	60.5 (419,168)	
**Previous CABG**	4.9 (5011)	2.9 (2166)	4.2 (10,572)	2.1 (583)	2.8 (713)	2.9 (1264)	4.5 (1050)	2.9 (6574)	3.5 (19,840)	17.4 (120,385)
**Previous PCI**	26.2 (26,644)	25.0 (18,601)	29.5 (74,856)	11.6 (3275)	17.4 (4447)	17.6 (7668)	23.2 (5456)	32.6 (74,166)	29.6 (169,503)	17.4 (120,385)
**GRACE risk score**										56.0 (388,271)
**Lowest (≤ 70)**	4.3 (2595)	4.3 (1965)	5.0 (7517)	0.8 (135)	1.0 (184)	1.5 (388)	2.4 (311)	11.5 (11,760)	7.1 (21,686)	
**Low (71–87)**	8.0 (4863)	7.3 (3309)	8.7 (13,252)	1.8 (288)	2.2 (385)	3.7 (1000)	5.5 (701)	16.1 (16,437)	11.0 (33,539)	
**Intermediate to high (≥ 88)**	87.7 (53,095)	88.4 (39,979)	86.3 (131,031)	97.4 (16,077)	96.8 (17,365)	94.8 (25,338)	92.1 (11,862)	72.5 (74,235)	81.9 (249,892)	
**Number of comorbidities**	2 (2–3)	2 (1–3)	1 (1–2)	3 (2–3)	3 (2–4)	2 (2–3)	3 (2–3)	N/A	1 (0–2)	7.9 (124,218)
**Total**	630,613	599,586	620,866	572,496	620,161	604,733	599,402	693,388	570,884	

*COPD* chronic obstructive pulmonary disease, *IMD* index of multiple deprivation (continuous), *AMI* acute myocardial infarction, *STEMI* ST-elevated myocardial infarction, *NSTEMI* non-ST-elevated myocardial infarction, *CABG* coronary artery bypass graft, *Primary PCI* primary percutaneous coronary intervention; Global Registry of Acute Coronary Events (GRACE) risk score (categorised into lowest [< 70], low [70 to 87], and intermediate-to-high risk [≥ 88]); missing column refers to the level of missing for each variable in the dataset

### Receipt of care

**Fig. 2:**
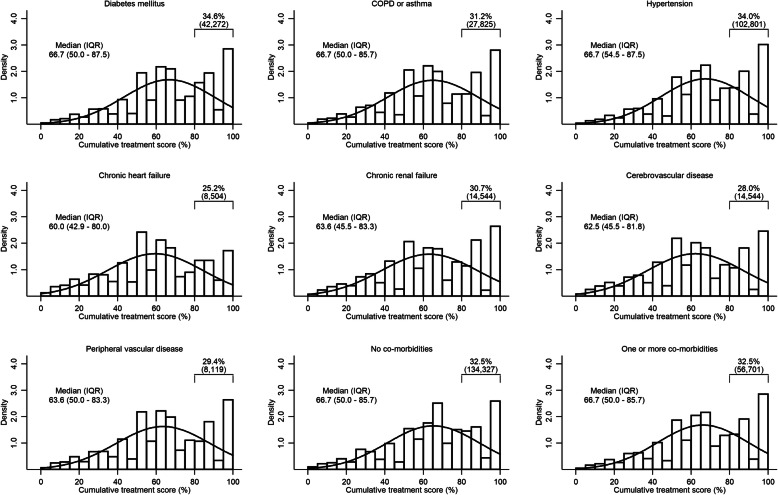
Treatment receipt ratio (total treatments received out of total eligible) by comorbidity group including those without comorbidity and those with one or more comorbidity. AMI patients presenting with the specified condition may have any of the other 6 comorbidities

The recorded use of ECG on admission was high across all comorbidities (≥ 93%), whilst the recorded provision of smoking cessation advice was consistently low (≤ 20%) (Table 2). The use of aspirin in the acute phase and some pharmacotherapies at time of hospital discharge (aspirin, ACEi/ARB and statins) was consistently high (> 70%); in contrast, the use of P2Y_12_ inhibitors and aldosterone antagonists at discharge was low across all comorbidities (< 45%). The use of an invasive coronary strategy was lower for those with comorbidities compared to those without, in particular for those with chronic heart failure (8864 [28.7%]), chronic renal failure (10,158 [36.2%]) and cerebrovascular disease (18,190 [62.5%]) compared to those without comorbidities (157,354 [57.7%]) (Table 2). AMI patients with only one comorbidity had similar rates of treatment receipt (Additional file 1: Table S3). Overall, patients with chronic heart failure had the lowest cumulative treatment score, receiving on average 60.0% of eligible treatments (IQR 42.9 to 80.0%) (Table 2). Patients with hypertension (34.6%) and diabetes mellitus (34.0%) had the highest proportion with cumulative treatment score of 80% or more, in contrast to patients with heart failure of whom only 25.2% had a cumulative treatment score of 80% or more (Fig. 2).

**Table 2:** Proportion of patients who received guideline-indicated treatment for which they were eligible according to comorbidity

Guideline-indicated care	Treatments received % (***N***)									
	Diabetes mellitus	COPD or asthma	Hypertension	Chronic heart failure	Chronic renal failure	Cerebrovascular disease	Peripheral vascular disease	Number of comorbidities		
**Pharmacological therapies**								0	1	2 ≤
Acute aspirin	89.8 (66,818)	90.7 (54,297)	91.3 (182,918)	87.2 (14,865)	87.9 (14,936)	88.5 (24,549)	88.2 (13,341)	86.4 (201,860)	91.0 (155,972)	90.1 (91,927)
Aspirin at discharge	86.2 (88,601)	86.1 (64,560)	86.8 (225,028)	81.5 (21,971)	84.2 (20,580)	84.0 (35,427)	84.5 (19,439)	85.0 (207,794)	86.4 (178,134)	85.8 (124,459)
P2Y_12_ inhibitors at discharge	40.5 (41,639)	38.6 (29,058)	39.8 (102,566)	31.9 (8669)	42.7 (10,609)	36.6 (15,726)	35.6 (8199)	38.1 (81,619)	38.3 (77,834)	39.4 (57,546)
β-blockers at discharge	72.0 (32,712)	56.7 (13,980)	74.9 (90,334)	71.4 (16,417)	67.6 (6994)	66.3 (12,766)	67.2 (7173)	75.2 (107,956)	74.2 (73,120)	69.2 (44,117)
ACE inhibitors/ARBs at discharge	82.6 (81,177)	78.2 (37,411)	80.6 (136,249)	77.5 (19,599)	72.0 (10,092)	74.9 (20,538)	76.9 (12,333)	74.7 (119,461)	77.8 (97,090)	80.7 (90,851)
Statins at discharge	84.8 (89,259)	83.5 (64,424)	85.0 (224,238)	77.0 (21,627)	80.7 (20,249)	81.5 (35,656)	83.0 (19,728)	82.6 (203,064)	84.1 (175,625)	83.8 (124,997)
**Aldosterone antagonists**										
At discharge	30.1 (520)	33.4 (117)	32.6 (519)	38.1 (408)	N/A	33.0 (114)	30.0 (68)	0.0 (0)	30.5 (151)	32.6
										6 (623)
At admission or discharge	34.5 (597)	37.7 (132)	36.9 (587)	42.3 (453)	N/A	36.4 (126)	35.2 (80)	0.0 (0)	33.3 (165)	36.8
										8 (708)
**Non-invasive therapies**										
Echocardiogram	56.7 (65,980)	56.6 (48,031)	57.5 (165,339)	54.2 (17,154)	56.7 (16,429)	53.5 (26,006)	57.7 (15,145)	52.0 (140,591)	55.7 (126,655)	57.1 (94,299)
ECG	95.5 (116,764)	95.7 (85,337)	96.0 (290,259)	93.8 (31,722)	96.1 (29,804)	95.6 (49,612)	95.1 (26,268)	93.9 (263,563)	95.6 (227,863)	95.7 (167,033)
Cardiac rehabilitation	73.2 (82,843)	73.8 (60,629)	75.6 (212,835)	62.8 (18,560)	66.0 (17,914)	67.5 (31,024)	72.2 (18,296)	74.8 (200,239)	76.0 (169,622)	72.0 (114,624)
Smoking cessation advice	16.7 (9464)	19.4 (9819)	17.4 (24,966)	8.3 (1248)	11.8 (1461)	14.0 (3326)	16.6 (2693)	20.2 (32,897)	18.4 (22,015)	15.9 (13,102)
Dietary advice	34.8 (40,140)	32.7 (27,584)	33.7 (96,587)	26.2 (8066)	35.0 (9796)	30.7 (14,630)	30.9(8052)	28.2 (76,044)	32.1 (72,932)	33.6 (54,844)
**Invasive therapies**										
Early invasive coronary procedures	51.2 (59,241)	48.6 (41,003)	54.4 (157,090)	28.7 (8864)	36.2 (10,158)	37.9 (18,190)	46.4 (12,145)	57.7 (157,354)	55.5 (126,868)	46.9 (76,944)
**Cumulative treatment score—median (IQR)**	66.7 (50.0–87.5)	66.7 (50.0–85.7)	66.7 (54.5–87.5)	60.0 (42.9–80.0)	63.6 (45.5–83.3)	62.5 (45.5–81.8)	63.6 (50.0–83.3)	66.7 (50.0–81.8)	66.7 (50.0–85.7)	66.7 (50.0–85.7)
**Percentage of optimal care receipt**	14.3 (14,449)	14.0 (12,512)	15.1 (45,657)	8.6 (2916)	13.2 (4101)	12.3 (6377)	13.2 (3655)	13.0 (36,413)	14.5 (34,599)	14.0 (24,355)

*COPD* chronic obstructive pulmonary disease, *ACE inhibitors* angiotensin-converting enzyme inhibitors, *ARB* angiotensin receptor blocker, *ECG* electrocardiogram, *N/A* not applicable as do not meet eligibility criteria; full eligibility criteria in appendices; smoking cessation advice given to those with smoking history; acute aspirin includes only those with direct admission who were not already on aspirin or contraindicated; early invasive coronary procedures include primary PCI or thrombolysis within 12 h for STEMI and coronary angiography or PCI within 72 h for NSTEMI patients; patients were classified as ineligible if a treatment was listed as contraindicated, not indicated, or not applicable; if the patient declined treatment as recorded in MINAP; or if the patient was hospitalised prior to the publication year of treatment recommendation in the guidelines

**Fig. 3:**
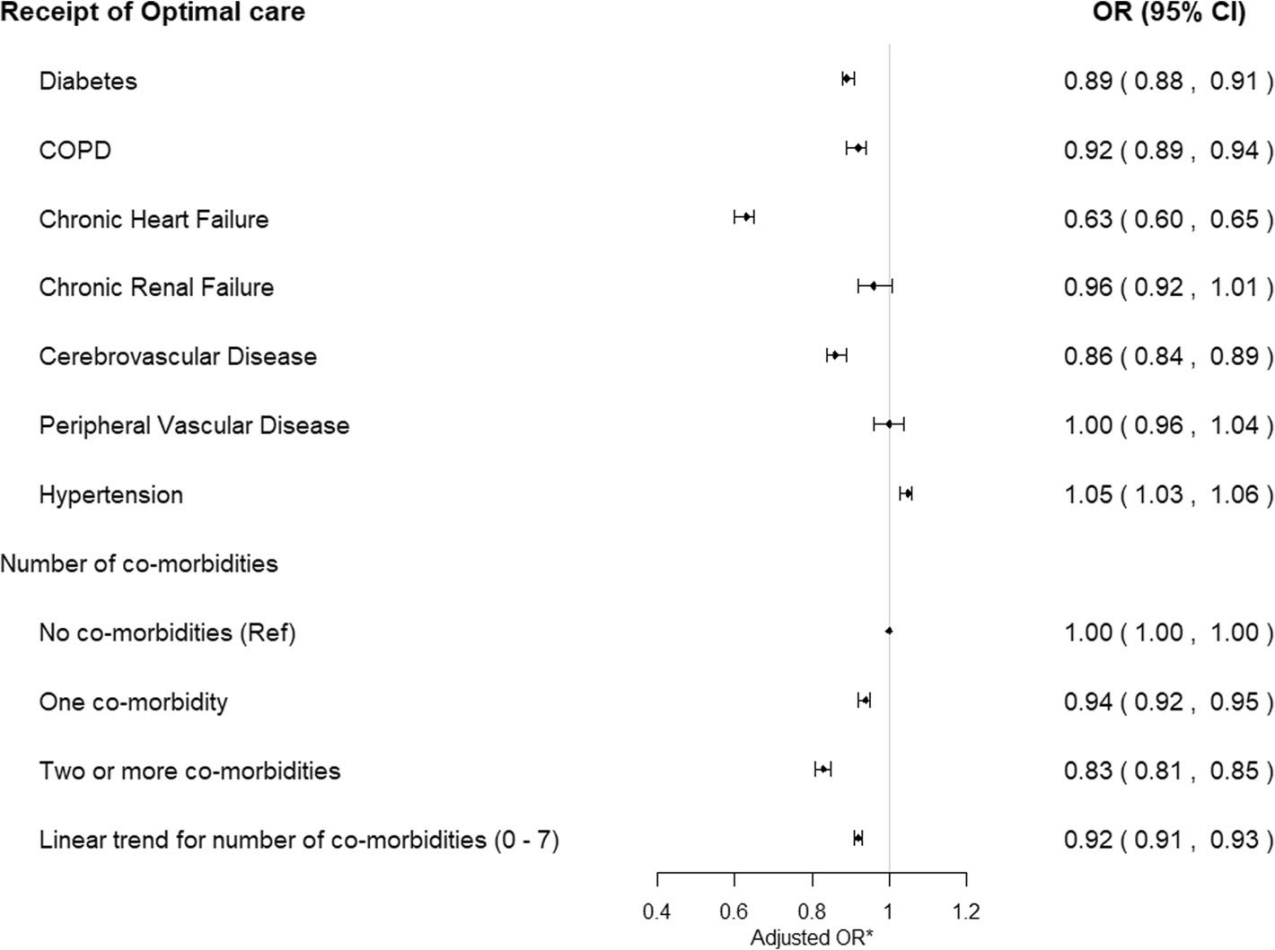
Association of comorbidities with receipt of optimal AMI care. Missing data multiply imputed. Patients with each of the chronic conditions may be subject to diagnosis of the 6 other chronic conditions. *Adjusted for GRACE risk, sex, year of diagnosis, smoking status, IMD score and all seven chronic conditions; multiple imputation by chained equations was used to produce 10 imputed datasets to minimise potential bias due to missing data. COPD, chronic obstructive pulmonary disease; IMD, index of multiple deprivation (continuous); Global Registry of Acute Coronary Events (GRACE) risk score; patients were classified as ineligible if a treatment was recorded in MINAP as contraindicated, not indicated, or not applicable; if the patient declined treatment; or if the patient was hospitalised prior to the guideline treatment recommendation

The presence of each comorbidity, with the exception of hypertension, renal failure and peripheral vascular disease, was associated with a reduced chance of receiving optimal care. The effect was most pronounced in those with chronic heart failure (OR 0.63, 95% CI 0.60 to 0.65), followed by cerebrovascular disease (OR 0.86, 95% CI 0.84 to 0.89) and diabetes mellitus (OR 0.89, 95% CI 0.88 to 0.91) (Fig. 3; Additional file 1: Table S4). Similarly, the presence of each comorbidity (with the exception of hypertension, chronic renal failure and peripheral vascular disease) was associated with an increased risk of receiving fewer guideline-recommended treatments. The greatest discrepancy was seen in patients with chronic heart failure (incidence risk ratio 0.94, 95% CI 0.94 to 0.95) (Additional file 1: Table S4). In patients with only hypertension (OR 0.98, 95% CI 0.96–1.00) or only peripheral vascular disease (OR 0.96, 95% CI 0.87–1.07), there was no significant difference in the receipt of optimal care compared to those with no comorbidities (Additional file 1: Table S5). Similar results were observed in the complete case analysis (Additional file 1: Table S6), in which optimal care receipt was similar in patients with peripheral vascular disease to those with no comorbidities (OR 1.03, 95% CI 0.99–1.08), whereas all other comorbidities were associated with a differential receipt of treatment (Additional file 1: Table S7).

### Receipt of care and long-term survival

**Fig. 4:**
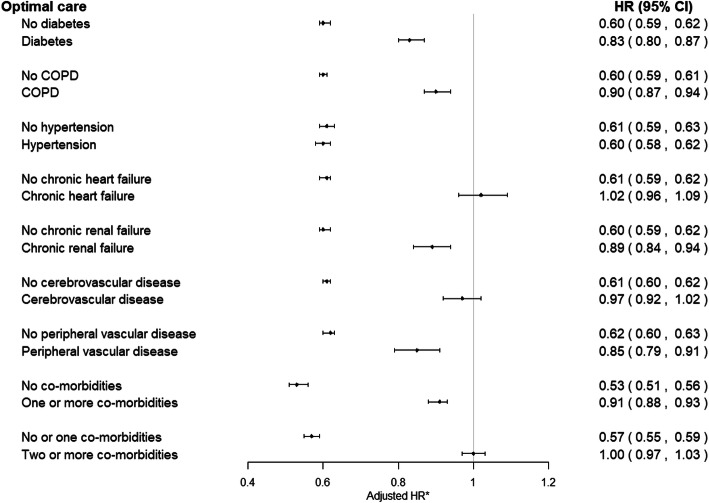
Association of receipt of optimal AMI care with long-term survival in the absence or presence of each comorbidity. Missing data multiply imputed. Patients with each of the chronic conditions may be subject to diagnosis of the 6 other chronic conditions. *Adjusted for GRACE risk, sex, year of diagnosis, smoking status, IMD score and all seven comorbidities; multiple imputation by chained equations was used to produce 10 imputed datasets to minimise potential bias due to missing data, and hazard ratios presented are pooled across all ten imputations. COPD, chronic obstructive pulmonary disease; IMD, index of multiple deprivation (continuous); Global Registry of Acute Coronary Events (GRACE) risk score

Over the 8.5-year follow-up period, the receipt of optimal care in patients without comorbidities was associated with a 47% reduced risk of death (adjusted HR 0.53, 95% CI 0.51 to 0.56) compared with a 9% reduced risk of death (adjusted HR 0.91, 95% CI 0.88 to 0.93) for patients who received optimal care and had at least one comorbidity (Fig. 4). Specifically, in patients with AMI who also had hypertension, diabetes mellitus, peripheral vascular disease, chronic renal failure and COPD or asthma, receipt of optimal AMI care was associated with a 40%, 17%, 15%, 11% and 10% relative reduced risk of death, respectively. However, for those with chronic heart failure and cerebrovascular disease and in patients with at least two comorbidities, the receipt of optimal AMI care was not associated with a significant change in survival (HR 1.02, 95% CI 0.96 to 1.09; HR 0.97, 95% CI 0.92 to 1.02; HR 1.00, 95% CI 0.97 to 1.03, respectively) (Fig. 4; Additional file 1: Table S8). In contrast, patients with heart failure and no other comorbidity had a 25% greater risk of mortality despite receiving optimal care (HR 1.25, 95% CI 1.03 to 1.52), and those with only diabetes, COPD, chronic renal failure, cerebrovascular disease and peripheral vascular disease showed no improvement in survival after receiving optimal care (Additional file 1: Table S9, Figure S1). In the complete case analysis, only in patients with heart failure was there no difference in survival despite receipt of optimal care (HR 1.01, 95% CI 0.94 to 1.09) (Additional file 1: Table S10).

**Fig. 5:**
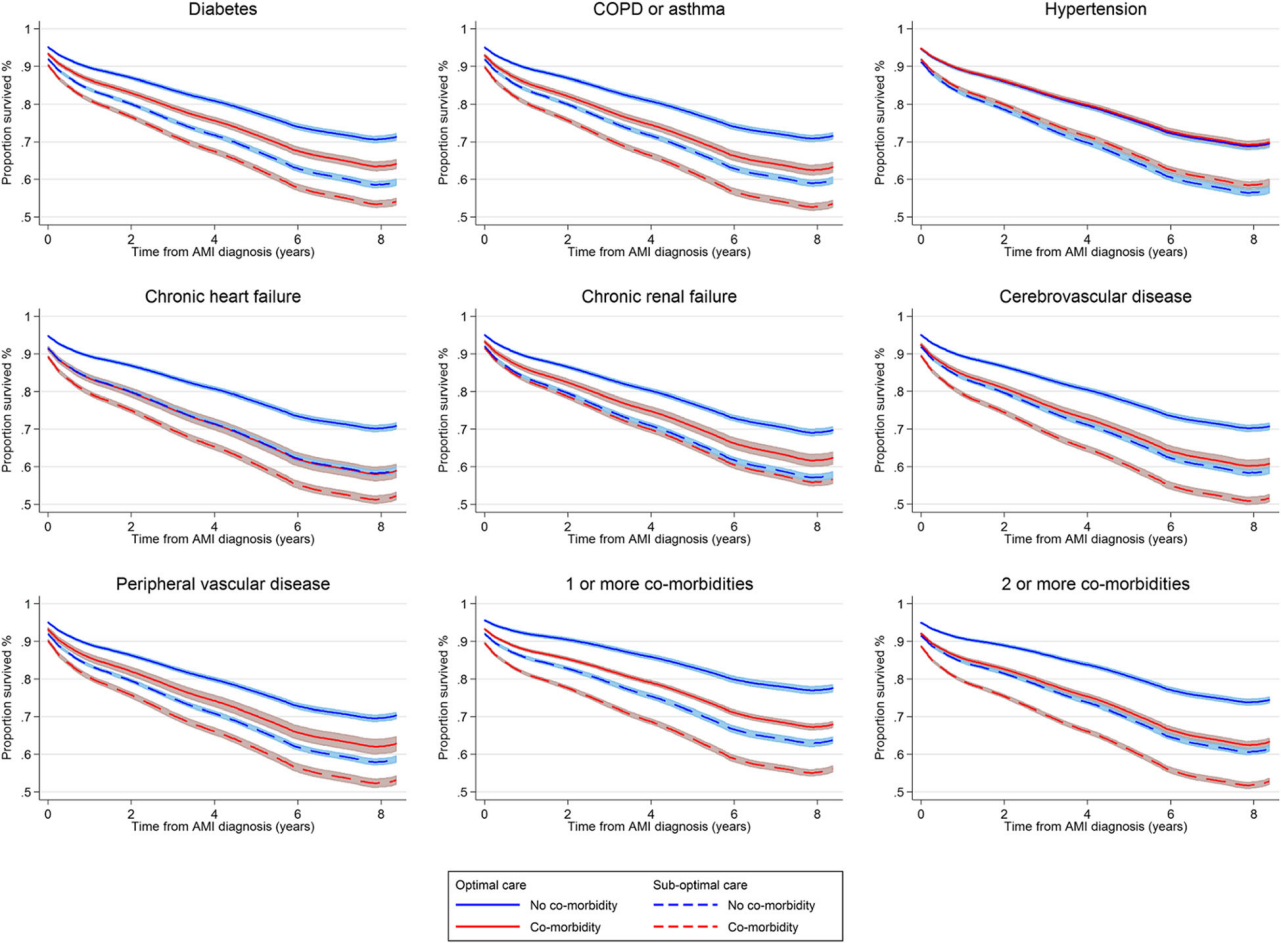
Survival graph of optimal AMI care vs suboptimal AMI care. *Adjusted for GRACE risk, sex, year of diagnosis, smoking status and IMD score

Long-term survival for AMI patients with diabetes mellitus, peripheral vascular disease, chronic renal failure and COPD or asthma was higher for those who received optimal care compared with those who did not (63.6% vs 53.6%; 62.1% vs 52.4%, 61.8% vs 56.0%, 62.6% vs 52.9%, respectively) (Fig. 5). For patients with chronic heart failure and cerebrovascular disease, and in those with two or more comorbidities, there was no difference in long-term survival between those who did and did not receive optimal care (58.2% vs 51.4%; 60.3% vs 51.0% and 62.6% vs 52.2%, respectively) (Fig. 5).

The magnitude and direction of the estimates were upheld in sensitivity analyses comparing the imputed data with a complete case analysis, with the exception of AMI with cerebrovascular disease and patients with two or more comorbidities (Additional file 1: Table S9, S11; Figure S2, S3).

## Discussion

In this nationwide study of nearly 700,000 people hospitalised with AMI, we have shown that the co-existence of diabetes mellitus, COPD or asthma, chronic heart failure and cerebrovascular disease is common and is inversely associated with receipt of optimal guideline-recommended care for AMI. The most pronounced difference in care provision was seen amongst those with chronic heart failure compared to those without. Overall, the receipt of optimal AMI care was associated with reduced mortality. However, in patients with chronic heart failure and cerebrovascular disease or those with two or more comorbidities, there was no significant improvement in survival despite receipt of optimal guideline-indicated AMI care.

Whilst there have been substantial improvements in long-term survival for patients with AMI, predominantly owing to an increased uptake of an invasive coronary strategy [20], the prognosis for patients with heart failure remains poor [26]. In this study, the long-term survival of patients with AMI was almost 50% lower if they had concomitant heart failure. These disadvantaged outcomes may relate to decreased provision or efficacy of therapy. In this study, we have shown that patients with chronic heart failure and cerebrovascular disease and those with two or more comorbidities had a 37%, 14% and 17% reduced chance of receiving optimal care in comparison to those without comorbidities. However, when patients with chronic heart failure, cerebrovascular disease or two or more comorbidities did receive optimal guideline-indicated AMI care, there was no evidence of a survival benefit compared with those who did not receive optimal guideline-indicated AMI care. There are many possible reasons for the lack of survival benefit seen amongst these comorbidity groups. In particular, evidence-based care for AMI is largely determined by randomised clinical trials that were optimised to focus on single diseases and single disease pathways, and the relative efficacy of the interventions in the presence of comorbidity is not known. Additionally, the prognosis of these AMI subgroups, in particular for patients with concomitant heart failure, is poor and may lack reversibility, and some of the current therapies may be poorly tolerated or lack benefit [1]. Data demonstrating the efficacy of novel therapies, such as sodium-glucose cotransporter 2 (SGLT2) inhibitors, in patients with heart failure and a reduced ejection fraction (even in a non-diabetic population) offers some grounds for optimism in this population [27]. In particular, our findings highlight the importance of developing research into new therapeutics which take into account the multimorbid nature of the majority of patients with AMI.

Our research identifies the overall low use of guideline-indicated therapies, including P_2_Y_12_ inhibitors for which the overall receipt of care was 39% and the use of invasive coronary strategies which was 29%, 38% and 47% amongst those with chronic heart failure and cerebrovascular disease and those with ≥ 2 comorbidities. We have previously reported on the overall low provision of care in detail [21, 24] and note that there are several explanations why this might be the case. The management of AMI is governed by international guidelines which are frequently updated [22, 28], and there is an underlying assumption that these are implemented into healthcare. However, as well as time lags associated with implementation, there are differences between international guidelines and national guidelines for the UK [29]—leading to inconsistency in implementation across the healthcare system. Moreover, the management of AMI is multifaceted, and this is particularly the case for people with comorbidities who may have competing healthcare needs, contraindications and differing priorities. For patients with comorbidities (and those with NSTEMI in particular, amongst whom comorbidity is more common), the approach to clinical management is heterogenous. Decisions regarding prescription or non-prescription of evidence-based medications, or to proceed to coronary angiography, are determined at the level of the physician in a non-emergent setting, and informed by the priorities and preferences of the patient. Finally, there is a known treatment-risk paradox with regard to the provision of invasive coronary procedures. Our previous work demonstrated that fewer than half of a predominantly (> 80%) high-risk NSTEMI population received invasive coronary procedures [20]. It is likely that patients with two or more comorbidities are amongst the highest-risk patients. The levels of care provision identified in this study are consistent with international comparisons, in which patients with AMI and heart failure are less likely to undergo reperfusion or be prescribed on aspirin on hospital discharge [30].

One strength of this study was the use of MINAP, which is the largest nationwide single healthcare system database of prospectively collected data on patients with AMI, with robust mortality data provided through linkage to the Office of National Statistics. Our analysis using imputed data allowed a more complete analytical approach in order to examine the effect of comorbidity states on receipt of optimal care, and the impact of this interaction on survival. To our knowledge, this is the first such analysis. However, we recognise the limitations of our work. We were reliant upon the accurate recording of data in MINAP. Whilst case ascertainment in MINAP is high, not all cases of NSTEMI in England and Wales are entered in the registry [19]. Moreover, we have relied on the accurate recording of comorbidities in MINAP and were limited to studying the set of conditions specifically collected as part of a patients’ medical history. Patients may have had additional comorbidities that could have influenced their treatment that we were unable to account for, and this may in part explain why the overall provision of invasive coronary angiography in patients without comorbidities appeared low (57%). We used a strict definition of optimal care based on an “all or none” approach, which could misclassify some patients for which data were missing. However, in addition to our multiple imputation approach (to minimise potential bias caused by missing data), we also conducted a sensitivity analyses using a cumulative treatment score to capture the variation in treatment provision, which showed consistent results. We did not have data on post-AMI hospital admissions; therefore, we were unable to account for competing events occurring within the follow-up period. We were not able to quantify uptake of cardiac rehabilitation, as only the presence of a referral to cardiac rehabilitation is recorded within MINAP. Moreover, our analyses were restricted to all-cause mortality; however, in the context of multimorbidity, this may be a more clinically useful measure than cause-specific mortality [31].

There is a degree of urgency to improving care for patients with multimorbidity, as there is clear evidence that population ageing and improved survival from acute illness have led to a substantial increase in the burden of comorbidity in patients with cardiovascular disease over recent decades [32]. There is a need for both increased recognition of the adverse prognostic implications of multimorbidity, but also a clear understanding of the clinical profiles of patients in whom current guideline-indicated therapy is less effective. This will enable clinicians to more accurately prognosticate, to inform shared decision-making and also to target future medical intervention to this vulnerable group.

## Conclusions

In this large, nationwide cohort study, we have investigated the association between comorbidities, receipt of care and clinical outcomes for patients with AMI. We showed that patients with heart failure, cerebrovascular disease, diabetes mellitus and COPD or asthma were less likely to receive guideline-recommended care. Whilst overall, guideline-indicated care was associated with improvements in mortality, this study found no effect in patients with heart failure or cerebrovascular disease.

These findings suggest that there is a need to improve adherence to guideline-recommended therapy in AMI patients with concomitant diabetes mellitus and COPD or asthma who were less likely to receive optimal care—but for whom prognosis could be improved if optimal care was provided. Whilst questions around development of future comorbidities following AMI remain unanswered, our findings do suggest there may be a need for the development of novel treatment pathways specifically aimed at improving outcomes for AMI patients with pre-existing heart failure or cerebrovascular disease.

## Supplementary information

**Additional file 1.**

## Data Availability

Data may be requested from the National Institute for Cardiovascular Outcomes Research (NICOR), https://www.nicor.org.uk/. Further details on data request applications may be found on https://www.nicor.org.uk/national-cardiac-audit-programme/myocardial-ischaemia-minap-heart-attack-audit/.
