# Supplementary material for: Association of treatments for acute myocardial infarction and survival for seven common comorbidity states: a nationwide cohort study
Source: BMC Med. 2020 Aug 24;18:231. doi: 10.1186/s12916-020-01689-5 (PMC7444071; doi:10.1186/s12916-020-01689-5)
Supplement: Supplementary file 1 — Additional file 1. [file 12916_2020_1689_MOESM1_ESM.docx]

**Table S1: AMI treatments and eligibility criteria as defined in analysis**

| **Acute myocardial infarction treatments and eligibility criteria** | | |
| --- | --- | --- |
| **Therapy** | **Eligibility Criteria** | **Opportunity Received** |
| **Pharmacological therapies** | | |
| **Aspirin** |  |  |
| **Acutely  (pre-hospital admission)** | All patients arriving via ambulance and not already on aspirin or contraindicated. | Aspiring given when arriving via ambulance and during admission. |
| **At Discharge** | All patients not already on aspirin or contraindicated. | If discharged on aspirin whilst not receiving before and during admission. |
| **P2Y_12_ inhibitor at discharge** | All patients not contraindicated and without high risk of bleeding. If discharged on thienopyridine or ticagrelor. | If discharged on thienopyridine or ticagrelor. |
| **β-Blocker at discharge** | All patients with reduced LV function, no heart failure diagnosis and no contraindications.  STEMI patients’ eligible if admitted after year 2011 and NSTEMI patients after 2006. | If discharged on beta blocker or oral beta blockers.  *NOTE: For UK guidelines everyone is eligible for β-Blockers.* |
| **ACE inhibitors/ARBs** | All patients with reduced LV function, no heart failure or diabetes diagnosis and no contraindications. | If discharged on ACEi/ARB. |
| **Statin** | All patients unless contraindicated. | Discharged on statin. |
| **Aldosterone antagonist/eplerenone** |  | |
| **At discharge** | Patients with depressed LV function (LVEF ≤35%) and either diabetes or heart failure, without significant renal dysfunction and already treated with ACE inhibitors and beta blockers  STEMI patients’ eligible if admitted after year 2011 and NSTEMI patients after 2006. | Aldosterone/ eplerenone given at discharge. |
| **In-hospital and at discharge** |  | Aldosterone/ eplerenone given either during admission or at discharge. |
| **Non-invasive therapies** | | |
| **Echocardiogram** | All patients unless not indicated. | If echocardiogram received in hospital or planned after discharge. |
| **12 lead ECG** |  |  |
| **Pre-Hospital** | All patients arriving via ambulance | If ECG administered |
| **In-Hospital** | All patients not already received ECG pre-hospital |  |
| **Referral for Cardiac Rehabilitation** | All patients eligible unless not indicated or declined by patient. | Cardiac rehabilitation referral made. |
| **Smoking Cessation Advice** | All patients eligible unless not applicable or non-smoker. | If smoking cessation given or planned in rehab. |
| **Dietary Advice** | All patients eligible if applicable. | If dietary advice given or planned in rehab. |
| **Early Invasive Procedures** |  |  |
| **STEMI** |  |  |
| **Primary Percutaneous Coronary Intervention (PCI) or Thrombolysis** | Eligible if not for one of the following reasons:  Risk of haemorrhage  Uncontrolled hypertension  Patient refused treatment | If PCI or thrombolysis performed within the first 12 hours |
| **NSTEMI** |  |  |
| **PCI or coronary angiography** | Eligible if diagnosis of NSTEMI including high risk patients which were classed as having any of the following:   - diagnosis of diabetes, renal failure or heart failure - previous PCI - previous CABG - poor ejection fraction - intermediate to high GRACE score - high risk of NSTEMI | If PCI or coronary angiography performed |

Patients were classified as ineligible if a treatment was listed as contraindicated, not indicated, not applicable, if the patient declined treatment as recorded in MINAP or if the patient was hospitalized prior to the publication year of treatment recommendation in the guidelines.

**Table S2.** Imputation model specification.

| **Variable** | **Variable Type** | **Imputation method** |
| --- | --- | --- |
| Age | Continuous, non-normal | Predictive mean matching |
| Index of multiple deprivation score | Continuous, non-normal | Predictive mean matching |
| Ethnicity | Categorical | Polytomous logistic regression |
| Sex | Binary | Logistic regression |
| Systolic blood pressure | Continuous, non-normal | Predictive mean matching |
| Heart rate | Continuous, non-normal | Predictive mean matching |
| Peak troponin | Continuous, non-normal | Predictive mean matching |
| Creatinine | Continuous, non-normal | Predictive mean matching |
| Loop diuretic | Binary | Logistic regression |
| Cardiac arrest | Binary | Logistic regression |
| ECG Appearance | Categorical | Polytomous logistic regression |
| Aspirin | Binary | Logistic regression |
| Beta-blocker | Binary | Logistic regression |
| Statin | Binary | Logistic regression |
| ACEi or ARBs | Binary | Logistic regression |
| P2Y_12_ inhibitors | Binary | Logistic regression |
| Aldosterone antagonist | Binary | Logistic regression |
| Coronary intervention | Categorical | Polytomous logistic regression |
| Diabetes mellitus | Binary | Logistic regression |
| Previous hypertension | Binary | Logistic regression |
| Elevated cholesterol | Binary | Logistic regression |
| Previous myocardial infarction | Binary | Logistic regression |
| Previous angina | Binary | Logistic regression |
| Peripheral vascular disease | Binary | Logistic regression |
| Cerebrovascular disease | Binary | Logistic regression |
| COPD or asthma | Binary | Logistic regression |
| Congestive renal failure | Binary | Logistic regression |
| Congestive cardiac failure | Binary | Logistic regression |
| Previous PCI | Binary | Logistic regression |
| Previous CABG | Binary | Logistic regression |
| Family history of chronic heart disease | Binary | Logistic regression |
| Smoking status | Binary | Logistic regression |
| Care by a cardiologist | Binary | Logistic regression |
| Year | Continuous | Predictor variable only |
| Admission diagnosis | Categorical | Predictor variable only |
| Timing of invasive coronary strategy | Continuous | Predictor variable only |
| Nelson-Aalen estimate of survival | Continuous | Predictor variable only |
| Censoring indicator | Binary | Predictor variable only |

**Table S3: AMI treatments for patients with AMI and one co-morbidity**

|  | **Treatments Received  %(N)** | | | | | | |
| --- | --- | --- | --- | --- | --- | --- | --- |
| **Guide-line indicated therapies** | **Diabetes** | **COPD/Asthma** | **Hypertension** | **CHF** | **CRF** | **CVD** | **PVD** |
| **Pharmacological therapies** | (28,504) | (31,699) | (154,880) | (5,368) | (3,217) | (10,208) | (4,426) |
| **Aspirin acutely** | 88.5 | 91.3 | 91.9 | 84.5 | 87.0 | 87.7 | 88.9 |
|  | (17,532) | (22,507) | 103,904) | (2,422) | 1,774) | (5,254) | 2,579) |
| **Aspirin at discharge** | 85.4 | 86.1 | 87.2 | 78.8 | 82.5 | 82.4 | 85.2 |
|  | (20,767) | (23,465) | (118,315) | (3,381) | (2,071) | (6,883) | (3,252) |
| **P2Y_12_ Inhibitors** | 39.1 | 37.1 | 39.3 | 26.8 | 38.3 | 32.5 | 32.0 |
|  | (9,321) | (10,021) | (52,450) | (1,152) | (980) | (2,722) | 1,188) |
| **β-blockers** | 72.7 | 56.2 | 78.1 | 68.7 | 62.8 | 63.5 | 70.8 |
|  | (8,412) | (4,990) | (52,435) | (2,662) | (639) | (2,586) | (1,396) |
| **ACE inhibitors/ARBs** | 80.1 | 75.4 | 79.0 | 72.9 | 58.3 | 64.9 | 73.5 |
|  | (18,703) | (11,574) | (58,691) | (2,967) | (561) | (2,897) | (1,697) |
| **Statins** | 83.5 | 83.3 | 85.3 | 70.4 | 76.5 | 77.9 | 83.4 |
|  | (20,585) | (23,128) | (116,962) | (3,107) | (1,945) | (6,651) | (3,247) |
| **Aldosterone antagonists** | 26.0 | N/A | N/A | 38.0 | N/A | N/A | N/A |
| **(discharge only)** | (80) | N/A | N/A | (71) | N/A | N/A | N/A |
| **Aldosterone antagonists** | 27.6 | N/A | N/A | 42.8 | N/A | N/A | N/A |
| **(admissions & discharge)** | (85) | N/A | N/A | (80) | N/A | N/A | N/A |
| **Non-invasive therapies** |  |  |  |  |  |  |  |
| **Echocardiogram** | 53.1 | 55.7 | 57.0 | 50.1 | 50.8 | 48.1 | 55.8 |
|  | (14,493) | (16,886) | (84,253) | (2,519) | (1,514) | (4,626) | (2,364) |
| **ECG** | 95.1 | 95.4 | 96.0 | 91.5 | 95.1 | 94.4 | 94.0 |
|  | (27,100) | (30,246) | (148,750) | (4,912) | (3,058) | (9,637) | (4,160) |
| **Cardiac rehabilitation** | 73.3 | 76.0 | 77.9 | 60.2 | 63.7 | 65.3 | 75.4 |
|  | (19,602) | (22,546) | (113,837) | (2,838) | (1,794) | (5,872) | (3,133) |
| **Smoking cessation advice** | 18.2 | 21.6 | 18.3 | 6.7 | 11.8 | 14.2 | 19.1 |
|  | (2,565) | (4,168) | (13,714) | (156) | (155) | (692) | (565) |
| **Dietary advice** | 33.2 | 31.7 | 32.9 | 21.6 | 30.5 | 26.4 | 27.1 |
|  | (9,013) | (9,565) | (48,786) | (1,064) | (886) | (2,473) | (1,145) |
| **Invasive therapies** |  |  |  |  |  |  |  |
| **Early invasive coronary procedures** | 53.6 | 52.3 | 59.2 | 25.7 | 34.9 | 35.9 | 52.1 |
|  | (14,526) | (15,895) | (88,519) | (1,278) | 1,023) | (3,405) | (2,222) |

Cases only exposed to one morbidity were considered

COPD, chronic obstructive pulmonary disease; CHF, chronic heart failure; CRF, chronic renal failure; CVD, cerebrovascular disease; PVD, peripheral vascular disease; ACE inhibitors, angiotensin-converting enzyme inhibitors; ARB, angiotensin receptor blocker; ECG, electrocardiogram; N/A, not applicable as do not meet eligibility criteria; Patients were classified as ineligible if a treatment was listed as contraindicated, not indicated, not applicable, if the patient declined treatment as recorded in MINAP or if the patient was hospitalized prior to the publication year of treatment recommendation in the guidelines.

**Table S4: Receipt of optimal AMI care for patients with AMI**

|  | **Model 1 (Unadjusted)** | | **Model 2 (age, sex, IMD adjusted)** | | **Model 3 (Full adjustment)** | |
| --- | --- | --- | --- | --- | --- | --- |
| **Optimal Care*** | **OR (95% CI)** | **P-Value** | **OR (95% CI)** | **P-Value** | **OR (95% CI)** | **P-Value** |
| **Diabetes** | 0.94  (0.93 - 0.96) | <0.001 | 0.99  (0.98 - 1.01) | 0.500 | 0.89  (0.88 - 0.91) | <0.001 |
| **COPD or asthma** | 0.89  (0.87 - 0.91) | <0.001 | 0.94  (0.93 - 0.96) | <0.001 | 0.92  (0.89 - 0.94) | <0.001 |
| **Chronic heart failure** | 0.51  (0.49 - 0.53) | <0.001 | 0.61  (0.58 - 0.63) | <0.001 | 0.63  (0.60 - 0.65) | <0.001 |
| **Chronic renal failure** | 0.86  (0.83 - 0.89) | <0.001 | 1.00  (0.98 - 0.98) | 0.963 | 0.96  (0.92 - 1.01) | 0.098 |
| **Cerebrovascular disease** | 0.76  (0.74 - 0.79) | <0.001 | 0.89  (0.87 - 0.92) | <0.001 | 0.86  (0.84 - 0.89) | <0.001 |
| **Peripheral vascular disease** | 0.83  (0.80 - 0.86) | <0.001 | 0.90  (0.86 - 0.93) | <0.001 | 1.00  (0.96 - 1.04) | 0.983 |
| **Hypertension** | 1.03  (1.02 - 1.05) | <0.001 | 1.15  (1.13 - 1.16) | <0.001 | 1.05  (1.03 - 1.06) | <0.001 |
| **Number of co-morbidities** |  | <0.001 |  | <0.001 |  | <0.001 |
| **No co-morbidities** | Ref |  | Ref |  | Ref |  |
| **One co-morbidity** | 0.91  (0.89 - 0.92) | <0.001 | 1.01  (1.00 - 1.03) | 0.119 | 0.94  (0.92 - 0.95) | <0.001 |
| **Two or more co-morbidities** | 0.83  (0.82 - 0.85) | <0.001 | 1.00  (0.98 - 1.02) | 0.964 | 0.83  (0.81 - 0.85) | <0.001 |
| **Linear trend for number of co-morbidities** | 0.93  (0.92 - 0.93) | <0.001 | 0.99  (0.99 - 1.00) | 0.069 | 0.92  (0.91 - 0.93) | <0.001 |
|  | **Model 1 (Unadjusted)** | | **Model 2 (age, sex, IMD adjusted)** | | **Model 3 (Full adjustment)** | |
| **Cumulative Treatment**^‡^ | **IRR (95% CI)** | **P-Value** | **IRR (95% CI)** | **P-Value** | **IRR (95% CI)** | **P-Value** |
| **Diabetes** | 0.99  (0.99 - 0.99) | <0.001 | 1.01  (1.00 - 1.01) | <0.001 | 0.99  (0.99 - 0.99) | <0.001 |
| **COPD or asthma** | 0.95  (0.94 - 0.95) | <0.001 | 0.96  (0.96 - 0.96) | <0.001 | 0.98  (0.98 - 0.99) | <0.001 |
| **Chronic heart failure** | 0.87  (0.87 - 0.88) | <0.001 | 0.92  (0.92 - 0.93) | <0.001 | 0.94  (0.94 - 0.95) | <0.001 |
| **Chronic renal failure** | 0.91  (0.91 - 0.92) | <0.001 | 0.95  (0.94 - 0.95) | <0.001 | 1.00  (0.99 - 1.00) | 0.548 |
| **Cerebrovascular disease** | 0.90  (0.90 - 0.90) | <0.001 | 0.94  (0.93 - 0.94) | <0.001 | 0.96  (0.96 - 0.97) | <0.001 |
| **Peripheral vascular disease** | 0.93  (0.93 - 0.94) | <0.001 | 0.95  (0.95 - 0.96) | <0.001 | 0.99  (0.99 - 1.00) | 0.053 |
| **Hypertension** | 1.00  (1.00 - 1.00) | 0.506 | 1.03  (1.03 - 1.03) | <0.001 | 1.01  (1.01 - 1.02) | <0.001 |
| **Number of co-morbidities** |  | <0.001 |  | <0.001 |  | <0.001 |
| **No co-morbidities** | Ref |  | Ref |  | Ref |  |
| **One co-morbidity** | 0.97  (0.97 - 0.97) | <0.001 | 1.00  (1.00 - 1.00) | 0.041 | 1.00  (0.99 - 1.00) | 0.004 |
| **Two or more co-morbidities** | 0.94  (0.93 - 0.94) | <0.001 | 0.98  (0.98 - 0.98) | <0.001 | 0.98  (0.97 - 0.98) | <0.001 |
| **Linear trend for number of co-morbidities** | 0.97  (2.60 - 2.63) | <0.001 | 0.99  (0.99 - 0.99) | <0.001 | 0.99  (0.99 - 0.99) | <0.001 |

Missing data multiply imputed

Model 1: unadjusted, model 2: adjusted for age, sex and IMD score; model 3: adjusted for GRACE risk, sex, year of diagnosis, smoking status, IMD score and all seven comorbidities; *Logistic regression for optimal care (all/none approach) reported as odds ratio (OR); ^‡^Poisson regression for number of treatments received (cumulative treatment) additionally adjusted for treatment eligibility reported as incidence risk ratio (IRR); Multiple imputation by chained equations was used to produce 10 imputed datasets to minimise potential bias due to missing data; COPD, chronic obstructive pulmonary disease; IMD, index of multiple deprivation (continuous); Global Registry of Acute Coronary Events (GRACE) risk score;

**Table S5: Receipt of optimal AMI care for patients AMI and one co-morbidity**

|  | **Model 1 (Unadjusted)** | | **Model 2 (age, sex, IMD adjusted)** | | **Model 3 (Full adjustment)** | |
| --- | --- | --- | --- | --- | --- | --- |
| **Optimal Care*** | **OR (95% CI)** | **P-Value** | **OR (95% CI)** | **P-Value** | **OR (95% CI)** | **P-Value** |
| **Diabetes** | 0.90  (0.87 - 0.94) | <0.001 | 0.97  (0.93 - 1.00) | 0.088 | 0.80  (0.77 - 0.84) | <0.001 |
| **COPD or asthma** | 0.96  (0.93 - 0.99) | 0.016 | 1.05  (1.02 - 1.09) | 0.004 | 0.91  (0.88 - 0.95) | <0.001 |
| **Chronic heart failure** | 0.43  (0.38 - 0.48) | <0.001 | 0.55  (0.49 - 0.62) | <0.001 | 0.49  (0.43 - 0.55) | <0.001 |
| **Chronic renal failure** | 0.78  (0.69 - 0.87) | <0.001 | 0.96  (0.86 - 1.07) | 0.485 | 0.84  (0.74 - 0.96) | 0.012 |
| **Cerebrovascular disease** | 0.72  (0.68 - 0.77) | <0.001 | 0.90  (0.85 - 0.96) | 0.002 | 0.77  (0.71 - 0.83) | <0.001 |
| **Peripheral vascular disease** | 0.91  (0.83 - 0.99) | 0.034 | 1.01  (0.92 - 1.10) | 0.898 | 0.96  (0.87 - 1.07) | 0.466 |
| **Hypertension** | 0.95  (0.93 - 0.96) | <0.001 | 1.07  (1.05 - 1.09) | <0.001 | 0.98  (0.96 - 1.00) | 0.074 |
|  | **Model 1 (Unadjusted)** | | **Model 2 (age, sex, IMD adjusted)** | | **Model 3 (Full adjustment)** | |
| **Cumulative Treatment**^‡^ | **IRR (95% CI)** | **P-Value** | **IRR (95% CI)** | **P-Value** | **IRR (95% CI)** | **P-Value** |
| **Diabetes** | 0.99  (0.98 - 0.99) | <0.001 | 1.00  (1.00 - 1.01) | 0.144 | 0.97  (0.97 - 0.98) | <0.001 |
| **COPD or asthma** | 0.96  (0.96 - 0.97) | <0.001 | 0.98  (0.98 - 0.99) | <0.001 | 0.98  (0.98 - 0.98) | <0.001 |
| **Chronic heart failure** | 0.84  (0.83 - 0.85) | <0.001 | 0.90  (0.89 - 0.91) | <0.001 | 0.90  (0.88 - 0.91) | <0.001 |
| **Chronic renal failure** | 0.88  (0.86 - 0.89) | <0.001 | 0.92  (0.91 - 0.93) | <0.001 | 0.98  (0.96 - 0.99) | <0.001 |
| **Cerebrovascular disease** | 0.87  (0.86 - 0.88) | <0.001 | 0.92  (0.91 - 0.93) | <0.001 | 0.93  (0.92 - 0.94) | <0.001 |
| **Peripheral vascular disease** | 0.95  (0.94 - 0.96) | <0.001 | 0.98  (0.96 - 0.99) | <0.001 | 0.98  (0.97 - 1.00) | 0.013 |
| **Hypertension** | 0.98  (0.98 - 0.98) | <0.001 | 1.01  (1.01 - 1.01) | <0.001 | 1.01  (1.00 - 1.01) | <0.001 |

Cases only exposed to one disease were considered

Model 1: unadjusted, model 2: adjusted for age, sex and IMD score; model 3: adjusted for GRACE risk, sex, year of diagnosis, smoking status, IMD score; *Logistic regression for optimal care (all/none approach) reported as odds ratio (OR); ^‡^Poisson regression for number of treatments received (cumulative treatment) additionally adjusted for treatment eligibility reported as incidence risk ratio (IRR); Multiple imputation by chained equations was used to produce 10 imputed datasets to minimise potential bias due to missing data; COPD, chronic obstructive pulmonary disease; IMD, index of multiple deprivation (continuous); Global Registry of Acute Coronary Events (GRACE) risk score;

**Table S6: Receipt of optimal AMI care for patients AMI and one co-morbidity**

|  | **Model 1 (Unadjusted)** | | **Model 2 (age, sex, IMD adjusted)** | | **Model 3 (Full adjustment)** | |
| --- | --- | --- | --- | --- | --- | --- |
| **Optimal Care*** | **OR (95% CI)** | **P-Value** | **OR (95% CI)** | **P-Value** | **OR (95% CI)** | **P-Value** |
| **Diabetes** | 1.00  (0.96 - 1.03) | 0.904 | 1.05  (1.02 - 1.09) | 0.006 | 0.76  (0.72 - 0.80) | <0.001 |
| **COPD or asthma** | 1.10  (1.06 - 1.14) | <0.001 | 1.20  (1.15 - 1.24) | <0.001 | 0.87  (0.83 - 0.91) | <0.001 |
| **Chronic heart failure** | 0.46  (0.42 - 0.52) | <0.001 | 0.62  (0.56 - 0.70) | <0.001 | 0.41  (0.35 - 0.47) | <0.001 |
| **Chronic renal failure** | 0.84  (0.75 - 0.94) | 0.002 | 1.05  (0.93 - 1.18) | 0.435 | 0.79  (0.68 - 0.92) | 0.002 |
| **Cerebrovascular disease** | 0.80  (0.75 - 0.85) | <0.001 | 1.02  (0.95 - 1.09) | 0.637 | 0.70  (0.65 - 0.76) | <0.001 |
| **Peripheral vascular disease** | 0.99  (0.90 - 1.08) | 0.742 | 1.08  (0.99 - 1.19) | 0.095 | 0.93  (0.83 - 1.05) | 0.260 |
| **Hypertension** | 1.23  (1.21 - 1.26) | <0.001 | 1.34  (1.31 - 1.36) | <0.001 | 0.98  (0.96 - 1.01) | 0.149 |
|  | **Model 1 (Unadjusted)** | | **Model 2 (age, sex, IMD adjusted)** | | **Model 3 (Full adjustment)** | |
| **Cumulative Treatment**^‡^ | **IRR (95% CI)** | **P-Value** | **IRR (95% CI)** | **P-Value** | **IRR (95% CI)** | **P-Value** |
| **Diabetes** | 1.01  (1.00 - 1.01) | <0.001 | 1.03  (1.02 - 1.03) | <0.001 | 0.96  (0.96 - 0.97) | <0.001 |
| **COPD or asthma** | 0.99  (0.99 - 1.00) | <0.001 | 1.02  (1.01 - 1.02) | <0.001 | 1.00  (0.99 - 1.00) | 0.311 |
| **Chronic heart failure** | 0.85  (0.84 - 0.86) | <0.001 | 0.93  (0.92 - 0.94) | <0.001 | 0.89  (0.88 - 0.91) | <0.001 |
| **Chronic renal failure** | 0.89  (0.87 - 0.90) | <0.001 | 0.95  (0.93 - 0.96) | <0.001 | 0.99  (0.97 - 1.02) | 0.496 |
| **Cerebrovascular disease** | 0.88  (0.88 - 0.89) | <0.001 | 0.95  (0.94 - 0.95) | <0.001 | 0.97  (0.96 - 0.98) | <0.001 |
| **Peripheral vascular disease** | 0.97  (0.96 - 0.98) | <0.001 | 0.99  (0.98 - 1.01) | 0.430 | 1.00  (0.98 - 1.02) | 0.785 |
| **Hypertension** | 1.04  (1.03 - 1.04) | <0.001 | 1.06  (1.06 - 1.07) | <0.001 | 1.01  (1.01 - 1.01) | <0.001 |

Complete case analysis

Cases only exposed to single disease considered

Model 1: unadjusted, model 2: adjusted for age, sex and IMD score; model 3: adjusted for GRACE risk, sex, year of diagnosis, smoking status, IMD score; *Logistic regression for optimal care (all/none approach) reported as odds ratio (OR); ^‡^Poisson regression for number of treatments received (cumulative treatment) additionally adjusted for treatment eligibility reported as incidence risk ratio (IRR); COPD, chronic obstructive pulmonary disease; IMD, index of multiple deprivation (continuous); Global Registry of Acute Coronary Events (GRACE) risk score;

**Table S7: Receipt of optimal AMI care for patients AMI**

|  | **Model 1 (Unadjusted)** | | **Model 2 (age, sex, IMD adjusted)** | | **Model 3 (Full adjustment)** | |
| --- | --- | --- | --- | --- | --- | --- |
| **Optimal Care*** | **OR (95% CI)** | **P-Value** | **OR (95% CI)** | **P-Value** | **OR (95% CI)** | **P-Value** |
| **Diabetes** | 1.05  (1.04 - 1.07) | <0.001 | 1.10  (1.08 - 1.12) | <0.001 | 0.88  (0.86 - 0.90) | <0.001 |
| **COPD or asthma** | 1.03  (1.01 - 1.05) | 0.012 | 1.10  (1.08 - 1.12) | <0.001 | 0.92  (0.89 - 0.94) | <0.001 |
| **Chronic heart failure** | 0.58  (0.56 - 0.60) | <0.001 | 0.68  (0.65 - 0.71) | <0.001 | 0.58  (0.55 - 0.61) | <0.001 |
| **Chronic renal failure** | 0.95  (0.92 - 0.99) | 0.005 | 1.08  (1.05 - 1.12) | <0.001 | 0.95  (0.90 - 0.99) | 0.030 |
| **Cerebrovascular disease** | 0.87  (0.85 - 0.89) | <0.001 | 1.01  (0.98 - 1.04) | 0.501 | 0.83  (0.80 - 0.86) | <0.001 |
| **Peripheral vascular disease** | 0.95  (0.92 - 0.99) | 0.010 | 1.03  (0.99 - 1.07) | 0.118 | 1.03  (0.99 - 1.08) | 0.171 |
| **Hypertension** | 1.22  (1.20 - 1.24) | <0.001 | 1.33  (1.31 - 1.35) | <0.001 | 1.03  (1.01 - 1.05) | 0.001 |
| **Number of co-morbidities** |  | <0.001 |  | <0.001 |  | <0.001 |
| **No co-morbidities** | Ref |  | Ref |  | Ref |  |
| **One co-morbidity** | 1.14  (1.12 - 1.16) | <0.001 | 1.25  (1.23 - 1.27) | <0.001 | 0.92  (0.90 - 0.94) | <0.001 |
| **Two or more co-morbidities** | 1.09  (1.07 - 1.11) | <0.001 | 1.28  (1.26 - 1.31) | <0.001 | 0.79  (0.77 - 0.81) | <0.001 |
| **Linear trend for number of co-morbidities** | 1.03  (1.02 - 1.03) | <0.001 | 1.09  (1.08 - 1.10) | <0.001 | 0.90  (0.90 - 0.91) | <0.001 |
|  | **Model 1 (Unadjusted)** | | **Model 2 (age, sex, IMD adjusted)** | | **Model 3 (Full adjustment)** | |
| **Cumulative Treatment**^‡^ | **IRR (95% CI)** | **P-Value** | **IRR (95% CI)** | **P-Value** | **IRR (95% CI)** | **P-Value** |
| **Diabetes** | 1.02  (1.02 - 1.02) | <0.001 | 1.03  (1.03 - 1.04) | <0.001 | 0.98  (0.98 - 0.98 | <0.001 |
| **COPD or asthma** | 0.98  (0.97 - 0.98) | <0.001 | 1.00  (0.99 - 1.00) | 0.012 | 1.00  (0.98 - 1.00) | 0.057 |
| **Chronic heart failure** | 0.90  (0.89 - 0.90) | <0.001 | 0.95  (0.95 - 0.96) | <0.001 | 0.93  (0.92 - 0.94) | <0.001 |
| **Chronic renal failure** | 0.93  (0.93 - 0.94) | <0.001 | 0.97  (0.97 - 0.98) | <0.001 | 1.00  (1.00 - 1.01) | 0.362 |
| **Cerebrovascular disease** | 0.92  (0.92 - 0.92) | <0.001 | 0.96  (0.96 - 0.97) | <0.001 | 0.98  (0.98 - 0.99) | <0.001 |
| **Peripheral vascular disease** | 0.96  (0.95 - 0.96) | <0.001 | 0.98  (0.98 - 0.98) | <0.001 | 1.01  (1.00 - 1.01) | 0.094 |
| **Hypertension** | 1.04  (1.03 - 1.04) | <0.001 | 1.06  (1.06 - 1.07) | <0.001 | 1.02  (1.01 - 1.02) | <0.001 |
| **Number of co-morbidities** |  | <0.001 |  | <0.001 |  | <0.001 |
| **No co-morbidities** | Ref |  | Ref |  | Ref |  |
| **One co-morbidity** | 1.01  (1.01 - 1.02) | <0.001 | 1.04  (1.04 - 1.05) | <0.001 | 1.00  (1.00 - 1.00) | 0.547 |
| **Two or more co-morbidities** | 0.99  (0.99 - 0.99) | <0.001 | 1.04  (1.04 - 1.04) | <0.001 | 0.98  (0.98 - 0.98) | <0.001 |
| **Linear trend for number of co-morbidities** | 0.99  (0.99 - 1.00) | <0.001 | 1.01  (1.01 - 1.01) | <0.001 | 0.99  (0.99 - 0.99) | <0.001 |

Complete case analysis

Model 1: unadjusted, model 2: adjusted for age, sex and IMD score; model 3: adjusted for GRACE risk, sex, year of diagnosis, smoking status, IMD score and all seven comorbidities ; *Logistic regression for optimal care (all/none approach) reported as odds ratio (OR); ^‡^Poisson regression for number of treatments received (cumulative treatment) additionally adjusted for treatment eligibility reported as incidence risk ratio (IRR); COPD, chronic obstructive pulmonary disease; IMD, index of multiple deprivation (continuous); Global Registry of Acute Coronary Events (GRACE) risk score;

**Table S8: Association of mortality with optimal AMI care for patients with AMI and the absence or presence of each co-morbidity**

|  | **Unadjusted** | | **Adjusted*** | |
| --- | --- | --- | --- | --- |
|  | **HR (95% CI)** | **P-value** | **HR (95% CI)** | **P-value** |
| **No diabetes** | 0.43 (0.42 - 0.44) | <0.001 | 0.60 (0.59 - 0.62) | <0.001 |
| **Diabetes** | 0.77 (0.74 - 0.80) | <0.001 | 0.83 (0.80 - 0.87) | <0.001 |
| **No COPD or asthma** | 0.44 (0.43 - 0.45) | <0.001 | 0.60 (0.59 - 0.61) | <0.001 |
| **COPD or asthma** | 0.80 (0.77 - 0.83) | <0.001 | 0.90 (0.87 - 0.94) | <0.001 |
| **No hypertension** | 0.42 (0.40 - 0.43) | <0.001 | 0.61 (0.59 - 0.63) | <0.001 |
| **Hypertension** | 0.59 (0.57 - 0.60) | <0.001 | 0.60 (0.58 - 0.62) | <0.001 |
| **No chronic heart failure** | 0.46 (0.45 - 0.47) | <0.001 | 0.61 (0.59 - 0.62) | <0.001 |
| **Chronic heart failure** | 1.85 (1.74 - 1.96) | <0.001 | 1.02 (0.96 - 1.09) | 0.454 |
| **No chronic renal failure** | 0.44 (0.43 - 0.45) | <0.001 | 0.60 (0.59 - 0.62) | <0.001 |
| **Chronic renal failure** | 1.61 (1.53 - 1.70) | <0.001 | 0.89 (0.84 - 0.94) | <0.001 |
| **No cerebrovascular disease** | 0.45 (0.44 - 0.46) | <0.001 | 0.61 (0.60 - 0.62) | <0.001 |
| **cerebrovascular disease** | 1.19 (1.13 - 1.25) | <0.001 | 0.97 (0.92 - 1.02) | 0.287 |
| **No peripheral vascular disease** | 0.45 (0.44 - 0.46) | <0.001 | 0.62 (0.60 - 0.63) | <0.001 |
| **Peripheral vascular disease** | 0.96 (0.90 - 1.03) | 0.331 | 0.85 (0.79 - 0.91) | <0.001 |
| **No co-morbidities** | 0.38 (0.37 - 0.40) | <0.001 | 0.53 (0.51 - 0.56) | <0.001 |
| **One or more co-morbidities** | 0.97 (0.94 - 0.99) | 0.015 | 0.91 (0.88 - 0.93) | <0.001 |
| **Zero or one co-morbidity** | 0.42 (0.41 - 0.43) | <0.001 | 0.57 (0.55 - 0.59) | <0.001 |
| **Two or more co-morbidities** | 1.07 (1.04 - 1.10) | <0.001 | 1.00 (0.97 - 1.03) | 0.786 |

Missing data multiply imputed

* Adjusted for GRACE risk, sex, year of diagnosis, smoking status, IMD score and all seven chronic conditions; Multiple imputation by chained equations was used to produce 10 imputed datasets to minimise potential bias due to missing data; COPD, chronic obstructive pulmonary disease; CVD, cerebrovascular disease; PVD, peripheral vascular disease; IMD, index of multiple deprivation (continuous); Global Registry of Acute Coronary Events (GRACE) risk score;

**Table S9: Association of mortality with optimal AMI care for patients with AMI and the absence or presence of each co-morbidity**

|  | **Unadjusted** | | **Adjusted*** | |
| --- | --- | --- | --- | --- |
|  | **HR (95% CI)** | **P-value** | **HR (95% CI)** | **P-value** |
| **No diabetes** | 0.37 (0.36 - 0.39) | <0.001 | 0.55 (0.52 - 0.58) | <0.001 |
| **Diabetes** | 0.88 (0.80 - 0.96) | 0.008 | 1.03 (0.93 - 1.13) | 0.541 |
| **No COPD** | 0.37 (0.36 - 0.39) | <0.001 | 0.55 (0.52 - 0.57) | <0.001 |
| **COPD** | 0.84 (0.77 - 0.91) | <0.001 | 0.99 (0.91 - 1.08) | 0.972 |
| **No hypertension** | 0.38 (0.37 - 0.40) | <0.001 | 0.55 (0.53 - 0.58) | <0.001 |
| **Hypertension** | 0.56 (0.54 - 0.59) | <0.001 | 0.60 (0.57 - 0.62) | <0.001 |
| **No chronic heart failure** | 0.37 (0.36 - 0.39) | <0.001 | 0.55 (0.52 - 0.58) | <0.001 |
| **Chronic heart failure** | 2.45 (2.04 - 2.94) | <0.001 | 1.25 (1.03 - 1.52) | 0.021 |
| **No chronic renal failure** | 0.37 (0.36 - 0.39) | <0.001 | 0.55 (0.53 - 0.58) | <0.001 |
| **Chronic renal failure** | 2.28 (1.90 - 2.74) | <0.001 | 1.07 (0.87 - 1.31) | 0.491 |
| **No cerebrovascular disease** | 0.37 (0.36 - 0.39) | <0.001 | 0.55 (0.52 - 0.58) | <0.001 |
| **Cerebrovascular disease** | 1.48 (1.31 - 1.69) | <0.001 | 1.14 (0.99 - 1.30) | 0.055 |
| **No peripheral vascular disease** | 0.37 (0.36 - 0.39) | <0.001 | 0.55 (0.53 - 0.58) | <0.001 |
| **Peripheral vascular disease** | 0.84 (0.66 - 1.05) | 0.139 | 0.96 (0.75 - 1.22) | 0.763 |

Missing data multiply imputed

Cases only exposed to single disease considered

* Adjusted for GRACE risk, sex, year of diagnosis, smoking status, IMD score; Multiple imputation by chained equations was used to produce 10 imputed datasets to minimise potential bias due to missing data; COPD, chronic obstructive pulmonary disease; CVD, cerebrovascular disease; PVD, peripheral vascular disease; IMD, index of multiple deprivation (continuous); Global Registry of Acute Coronary Events (GRACE) risk score;

|  | **Unadjusted** | | **Adjusted*** | |
| --- | --- | --- | --- | --- |
|  | **HR (95% CI)** | **P-value** | **HR (95% CI)** | **P-value** |
| **No diabetes** | 0.43 (0.42 - 0.44) | <0.001 | 0.57 (0.56 - 0.59) | <0.001 |
| **Diabetes** | 0.76 (0.73 - 0.79) | <0.001 | 0.81 (0.77 - 0.84) | <0.001 |
| **No COPD** | 0.43 (0.42 - 0.44) | <0.001 | 0.58 (0.56 - 0.59) | <0.001 |
| **COPD** | 0.80 (0.77 - 0.83) | <0.001 | 0.86 (0.82 - 0.90) | <0.001 |
| **No hypertension** | 0.41 (0.40 - 0.42) | <0.001 | 0.57 (0.54 - 0.59) | <0.001 |
| **Hypertension** | 0.57 (0.55 - 0.58) | <0.001 | 0.56 (0.54 - 0.58) | <0.001 |
| **No chronic heart failure** | 0.45 (0.44 - 0.46) | <0.001 | 0.58 (0.56 - 0.59) | <0.001 |
| **Chronic heart failure** | 1.83 (1.72 - 1.94) | <0.001 | 1.01 (0.94 - 1.09) | 0.775 |
| **No chronic renal failure** | 0.33 (0.32 - 0.34) | <0.001 | 0.58 (0.56 - 0.59) | <0.001 |
| **Chronic renal failure** | 1.97 (1.80 - 2.15) | <0.001 | 0.82 (0.77 - 0.87) | <0.001 |
| **No cerebrovascular disease** | 0.44 (0.43 - 0.45) | <0.001 | 0.58 (0.56 - 0.59) | <0.001 |
| **Cerebrovascular disease** | 1.18 (1.12 - 1.24) | <0.001 | 0.92 (0.87 - 0.98) | 0.007 |
| **No peripheral vascular disease** | 0.44 (0.44 - 0.45) | <0.001 | 0.59 (0.57 - 0.60) | <0.001 |
| **Peripheral vascular disease** | 0.97 (0.91 - 1.04) | 0.453 | 0.83 (0.77 - 0.90) | <0.001 |
| **No co-morbidities** | 0.34 (0.33 - 0.36) | <0.001 | 0.49 (0.47 - 0.52) | <0.001 |
| **One or more co-morbidities** | 0.83 (0.81 - 0.85) | <0.001 | 0.84 (0.81 - 0.87) | <0.001 |
| **Zero or one co-morbidity** | 0.40 (0.39 - 0.41) | <0.001 | 0.53 (0.51 - 0.55) | <0.001 |
| **Two or more co-morbidities** | 1.02 (0.99 - 1.05) | 0.126 | 0.93 (0.90 - 0.96) | <0.001 |

**Table S10: Association of mortality with optimal AMI care for patients with AMI and the absence or presence of each co-morbidity**

Complete case analysis

* Adjusted for GRACE risk, sex, year of diagnosis, smoking status, IMD score and all seven chronic conditions; Multiple imputation by chained equations was used to produce 10 imputed datasets to minimise potential bias due to missing data; COPD, chronic obstructive pulmonary disease; CVD, cerebrovascular disease; PVD, peripheral vascular disease; IMD, index of multiple deprivation (continuous); Global Registry of Acute Coronary Events (GRACE) risk score;

**Table S11: Association of mortality with optimal AMI care for patients with AMI and the absence or presence of each co-morbidity**

|  | **Unadjusted** | | **Adjusted*** | |
| --- | --- | --- | --- | --- |
|  | **HR (95% CI)** | **P-value** | **HR (95% CI)** | **P-value** |
| **No diabetes** | 0.34 (0.33 - 0.36) | <0.001 | 0.51 (0.48 - 0.54) | <0.001 |
| **Diabetes** | 0.80 (0.73 - 0.87) | <0.001 | 0.94 (0.84 - 1.05) | 0.296 |
| **No COPD** | 0.34 (0.33 - 0.36) | <0.001 | 0.50 (0.48 - 0.53) | <0.001 |
| **COPD** | 0.74 (0.68 - 0.80) | <0.001 | 0.89 (0.81 - 0.99) | 0.024 |
| **No hypertension** | 0.34 (0.33 - 0.36) | <0.001 | 0.51 (0.48 - 0.54) | <0.001 |
| **Hypertension** | 0.47 (0.45 - 0.49) | <0.001 | 0.54 (0.51 - 0.57) | <0.001 |
| **No chronic heart failure** | 0.34 (0.33 - 0.36) | <0.001 | 0.51 (0.48 - 0.54) | <0.001 |
| **Chronic heart failure** | 2.16 (1.81 - 2.59) | <0.001 | 1.19 (0.95 - 1.48) | 0.124 |
| **No chronic renal failure** | 0.34 (0.33 - 0.36) | <0.001 | 0.52 (0.49 - 0.55) | <0.001 |
| **Chronic renal failure** | 2.04 (1.70 - 2.44) | <0.001 | 0.85 (0.68 - 1.06) | 0.141 |
| **No cerebrovascular disease** | 0.34 (0.33 - 0.36) | <0.001 | 0.51 (0.48 - 0.54) | <0.001 |
| **Cerebrovascular disease** | 1.29 (1.14 - 1.47) | <0.001 | 1.04 (0.90 - 1.21) | 0.575 |
| **No peripheral vascular disease** | 0.34 (0.33 - 0.36) | <0.001 | 0.51 (0.48 - 0.54) | <0.001 |
| **Peripheral vascular disease** | 0.72 (0.57 - 0.91) | 0.006 | 0.87 (0.66 - 1.13) | 0.293 |

Cases only exposed to single disease considered

* Adjusted for GRACE risk, sex, year of diagnosis, smoking status, IMD score; COPD, chronic obstructive pulmonary disease; CVD, cerebrovascular disease; PVD, peripheral vascular disease; IMD, index of multiple deprivation (continuous); Global Registry of Acute Coronary Events (GRACE) risk score;

**Figure S1: Association of mortality with optimal AMI care for patients with AMI and the absence or presence of a single co-morbidity**


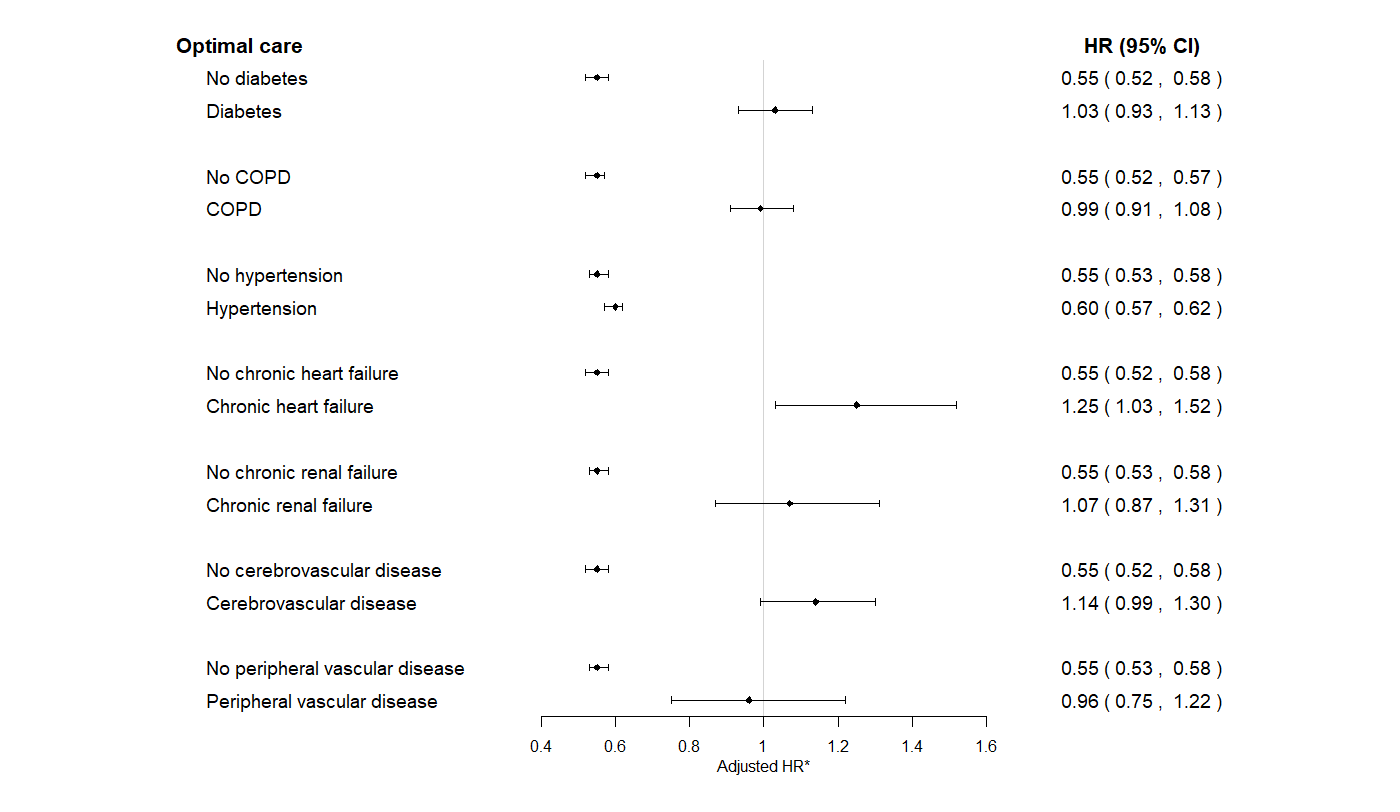


Missing data multiply imputed. Cases only exposed to single disease considered

* Adjusted for GRACE risk, sex, year of diagnosis, smoking status, IMD score and all seven chronic conditions; Multiple imputation by chained equations was used to produce 10 imputed datasets to minimise potential bias due to missing data; COPD, chronic obstructive pulmonary disease; IMD, index of multiple deprivation (continuous); Global Registry of Acute Coronary Events (GRACE) risk score;

**Figure S2: Association of mortality with optimal AMI care for patients with AMI and the absence or presence of each co-morbidity**


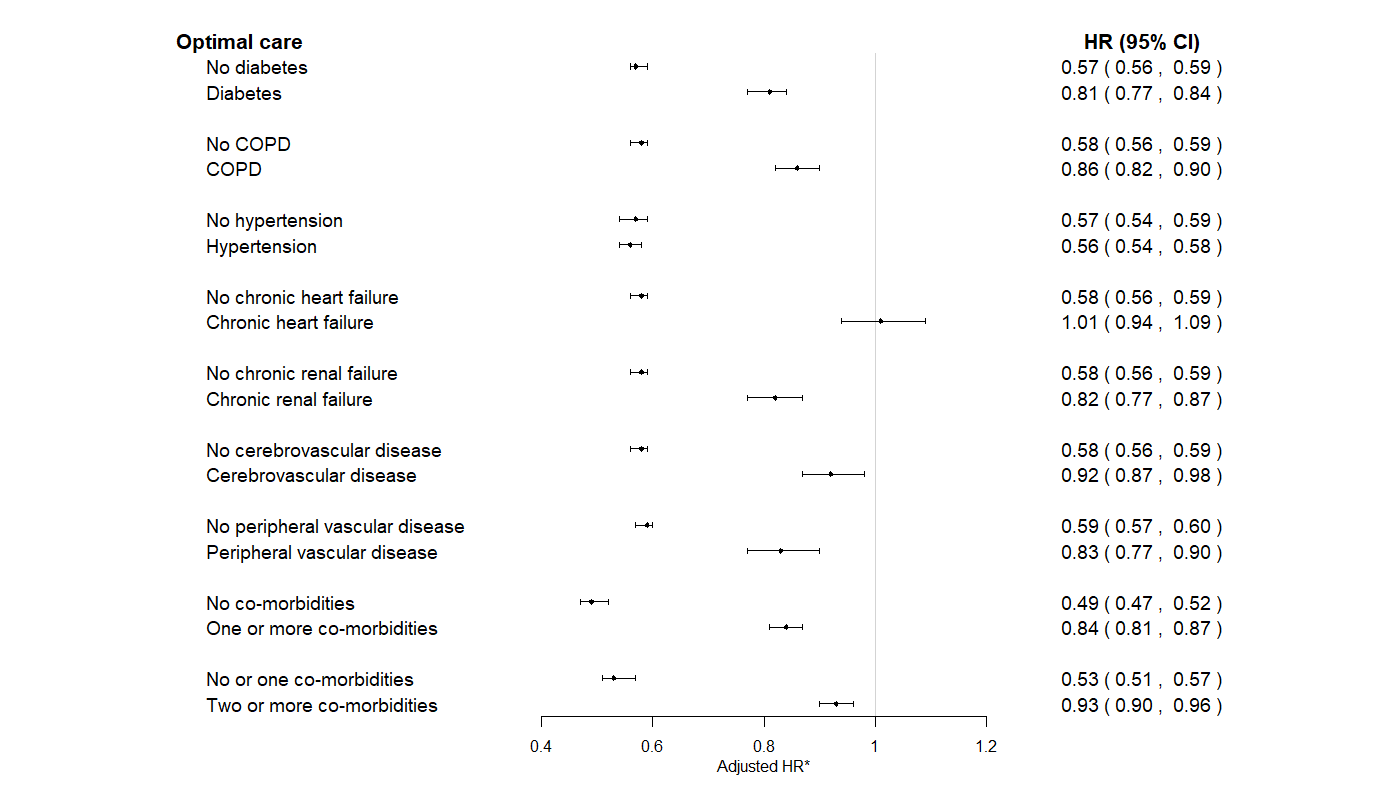


Complete case analysis

* Adjusted for GRACE risk, sex, year of diagnosis, smoking status, IMD score and all seven chronic conditions; COPD, chronic obstructive pulmonary disease; IMD, index of multiple deprivation (continuous); Global Registry of Acute Coronary Events (GRACE) risk score;

**Figure S3: Association of mortality with optimal AMI care for patients with AMI and the absence or presence of a single co-morbidity**


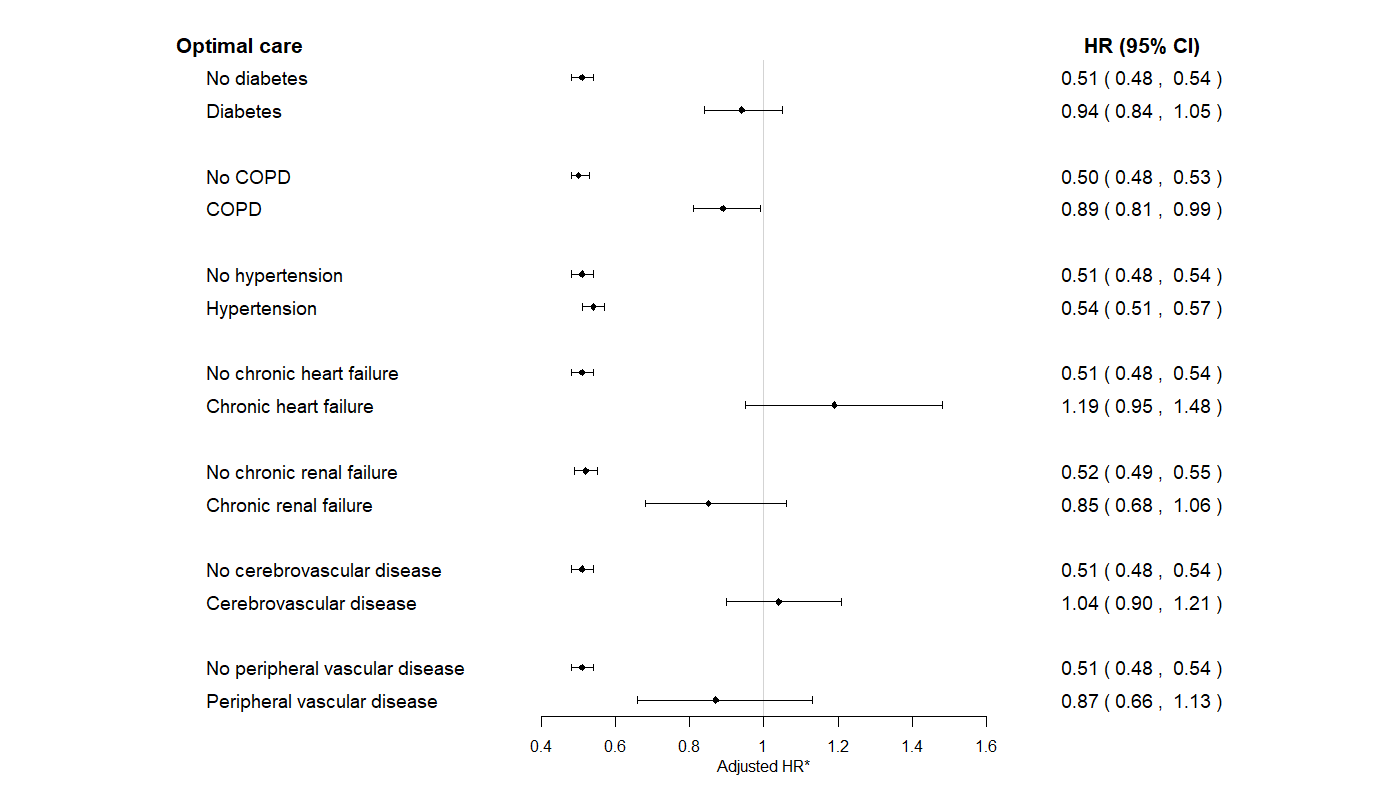


Complete case analysis. Cases only exposed to single disease considered

* Adjusted for GRACE risk, sex, year of diagnosis, smoking status, IMD score; COPD, chronic obstructive pulmonary disease; IMD, index of multiple deprivation (continuous); Global Registry of Acute Coronary Events (GRACE) risk score;
